# Will you be happy for me? Associations between self-reported, perceived, and observed responses to positive events and sexual well-being in cohabiting couples

**DOI:** 10.1177/02654075221080581

**Published:** 2022-03-28

**Authors:** Myriam Bosisio, Natalie O. Rosen, Justin Dubé, Marie-Pier Vaillancourt-Morel, Marie-Ève Daspe, Sophie Bergeron

**Affiliations:** 1 Department of Psychology, 5622 Université de Montréal, Montreal, Canada; 2 Departments of Psychology and Neuroscience, 3688 Dalhousie University, Halifax, Canada; 3 Department of Psychology, 14847 Université du Québec à Trois-Rivières, Trois-Rivieres, Canada

**Keywords:** Responses to capitalization attempts, responsiveness, positive event, sexual satisfaction, sexual distress, sexual function, sexual well-being

## Abstract

Whereas greater levels of intimacy have been shown to promote couples’ sexual well-being, recent theories suggest that satisfying sex is maintained via the capacity to encourage the partner’s individuality, while remaining intimately connected. Responses to capitalization attempts (i.e., the disclosure of a positive personal event) provide an opportunity to strengthen both the couple’s intimacy and each partner’s autonomy. Although responses to capitalization attempts are linked to couples’ greater relationship adjustment, very little is known about their relation to couples’ sexual well-being. The aim of this study was to examine the associations between self-reported, perceived, and observed responses to capitalization attempts and sexual satisfaction, sexual distress, and sexual function in 151 cohabiting couples who participated in a filmed discussion in the laboratory. They also completed self-report questionnaires pertaining to their responsiveness and to that of the partner during the discussion, as well as their sexual well-being. Results indicated that one’s higher levels of self-reported and partner-perceived active–constructive responses (enthusiasm/elaboration) during the discussion were associated with one’s own greater sexual satisfaction. Higher levels of perceived passive–constructive responses (quiet but interested) from one’s partner were associated with one’s own lower sexual satisfaction, and one’s higher levels of self-reported and perceived passive–destructive responses (lack of interest/self-focus) were associated with one’s own greater sexual distress. Finally, higher levels of observed active–destructive responses (undermines/denies the positive nature of the event) were associated with one’s own lower sexual function, while in women, they were associated with their lower sexual satisfaction. Findings contribute to a growing body of literature underscoring the importance of intimacy for sexual well-being in long-term relationships.

## Introduction

Couples’ sexual well-being tends to decline over time (Gunst et al., 2017; Quinn-Nilas, 2020). This is concerning, given that the quality of a couple’s sexuality is associated with several individual and relational benefits (Acevedo & Aron, 2009; Joel et al., 2020). There is growing interest in identifying positive interpersonal correlates of couples’ sexual well-being (e.g., Muise et al., 2016), although most studies to date involved retrospective self-reports (e.g., Dewitte & Mayer, 2018; Štulhofer et al., 2014). Among those, intimacy (i.e., emotional closeness or feeling understood, validated, and cared for by the partner following self-disclosure; Reis & Shaver, 1988) has received extensive empirical support (e.g., Bois et al., 2016; Rubin & Campbell, 2012). However, some argue that too much emotional closeness, to a point of emotional fusion, hinders the quality of couples’ sexuality (e.g., Sims & Meana, 2010). The *Sexual Crucible Model* suggests that true intimacy includes differentiation (i.e., the ability to see oneself as separate and distinct within the relationship), which is thought to promote couples’ sexual well-being (Schnarch, 2009). Responses to capitalization attempts, in which there is a disclosure of a positive personal event that does not involve the partner, would be an opportunity to demonstrate that ability to acknowledge the partner’s individuality, while remaining intimately connected (Gable et al., 2006; Hadden et al., 2015; Reis et al., 2010). Measuring moment-to-moment responses to capitalization attempts in a laboratory setting could provide crucial answers concerning the role of intimacy in couples’ sexual well-being. The aim of this study was to examine the associations between self-reported, perceived, and observed responses to capitalization attempts in the laboratory and sexual satisfaction, sexual distress, and sexual function in cohabiting couples.

## Decline in sexual well-being

Over time, most couples experience a decline in their sexual well-being, including sexual function and satisfaction (Impett et al., 2008; Murray & Milhausen, 2012; Quinn-Nilas, 2020). A 7-year longitudinal study using two time points found that women reported lower desire, arousal, lubrication, and satisfaction at Time 2 compared with Time 1 (Gunst et al., 2017). Schmiedeberg and Schröder (2016) showed that there was a steady decline in sexual satisfaction over a 3-year period (three time points) among individuals in committed relationships. This result is in line with those of two eight-wave longitudinal studies spanning the first four–five years of 207 marriages, showing that sexual frequency and sexual satisfaction declined over that period for newlyweds couples (McNulty et al., 2016). Although these studies were conducted almost exclusively among heterosexual individuals or couples, Blumstein and Schwartz’s (1983) study suggests similar declines in sexual frequency among gay and lesbian couples.

This decline is concerning given that couples’ sexual well-being is associated with greater relationship and life satisfaction, relationship stability, and psychological and physical health (Acevedo & Aron, 2009; Mark, 2012; Regan, 2000). Conversely, experiencing a sexual problem commonly leads to relational conflict and distress (Metz & Epstein, 2002; Rosen et al., 2017, 2019). However, only recently have studies begun to focus on protective interpersonal factors for couples’ sexual well-being. Intimacy figures prominently among the theory-driven protective factors examined to date.

## Intimacy and sexual well-being

According to the *Interpersonal Process Model of Intimacy* (Reis & Shaver, 1988), intimacy refers to an emotional closeness in which partners both feel understood, validated, and cared for following self-disclosure. Intimacy is associated with greater sexual satisfaction and function, and less sexual distress for the self and the partner in couples coping with pain during sexual activities, according to dyadic observational and daily diary studies (Bergeron et al., 2021; Bois et al., 2016). In experimental, longitudinal and daily diary studies among community couples, intimacy was associated with greater sexual desire, frequency, and satisfaction (Dewitte & Mayer, 2018; Mizrahi et al., 2018; Rubin & Campbell, 2012). Among those studies, a handful found that intimacy was more strongly associated with women’ sexuality than men’s (e.g., Birnbaum et al., 2016; Dewitte & Mayer, 2018). In addition, two cross-sectional studies conducted among men indicated that when they reported higher levels of intimacy with their partner, they also reported higher sexual satisfaction, desire, and lower levels of sexual difficulties (Štulhofer et al., 2013, 2014). Thus, findings to date suggest that greater intimacy is associated with greater sexual well-being and less sexual difficulties in both clinical and community couples. However, not all studies used theory-driven measures of intimacy and most used retrospective self-reports (see Bois et al., 2016, for an exception), which may lead to recall biases. Importantly, results from a couple observation study suggest that intimacy can be translated into specific observable behaviors (Collins & Feeney, 2000). Hence, observational designs offer a unique opportunity to study moment-to-moment intimacy processes that cannot be captured through self-report measures.

## Intimacy and differentiation

Despite the evidence supporting a positive association between intimacy and sexual well-being, authors suggest that too much emotional proximity (e.g., to a point of being emotionally merged or poorly differentiated) could be harmful for sexual well-being (Perel, 2007; Schnarch, 2009). The *Sexual Crucible Model* suggests that true intimacy derives from each partner’s capacity to maintain a personal identity and recognize the other as being different from the self, while simultaneously feeling connected to each other (Schnarch, 2009). This ability would allow the couple to establish the necessary space to bring novelty and vitality to their union and tolerate the vulnerability and insecurity inherent to sexual pleasure, thereby fostering sexual well-being (Kuten, 1976; Perel, 2007; Schnarch, 2009). However, these theories have received little support to date. Results from cross-sectional, self-report studies conducted among individuals suggest that the inability to maintain a sense of self in the presence of intimate others was the strongest predictor of sexual problems (Burri et al., 2014) and that differentiation of the self was associated with greater sexual satisfaction and desire (Timm & Keiley, 2011; Ferreira et al., 2014). The literature on self-expansion (i.e., expanding one’s sense of self through novel, exciting, and broadening activities) is also noteworthy in that it underlines the importance of novelty and vitality for couples’ sexual well-being. In a cross-sectional, self-report study among women diagnosed with low sexual desire and their partners, higher levels of self-expansion activities with the partner were associated with greater sexual satisfaction for both partners, higher desire for women, and lower sexual distress for partners (Raposo et al., 2020). A dyadic daily diary study among community couples showed that within-person increases in daily personal self-expansion (without the partner) were associated with greater sexual passion. However, experiencing chronically high levels of personal self-expansion in ways that were not shared with a romantic partner was associated with poorer intimacy, and in turn, less sexual passion (Carswell et al., 2021). These results support the idea that both the feelings of individuality and togetherness are important for sexual well-being (Schnarch, 2009).

## Responses to capitalization attempts

How romantic partners respond to capitalization attempts offers the potential for couples to encourage the partner’s individuality, while feeling connected to each other. Capitalization attempts refer to the process of disclosing a positive event to someone to gain additional benefits from it (Gable et al., 2006; Langston, 1994). For example, a positive event can be getting a good grade, talking to a childhood friend, or getting a promotion at work. Indeed, capitalizing on positive events (sharing good news) has been linked to increases in positive affect and well-being independently of the positive events themselves (Gable et al., 2004). However, this effect depends on the responses of the person with whom the events are shared. Most of the time, these are loved ones, such as the romantic partner (Gable et al., 2006). Researchers have identified four types of responses following the disclosure of a positive event: active–constructive (e.g., enthusiasm and elaboration), passive–constructive (e.g., quiet but attentive and interested), active–destructive (e.g., undermines the event or denies its positive nature), and passive–destructive (e.g., lack of interest and self-focus; Gable et al., 2004). When sharing a positive event that does not include the partner (e.g., receiving a positive evaluation at work), the partner’s active–constructive response is the only one considered responsive. This response provides an opportunity to obtain and convey understanding and validation while recognizing and accepting the partner’s individuality, thereby strengthening the couple’s intimacy (Gable et al., 2006; Hadden et al., 2015; Reis et al., 2010). In self-report and observational studies among community couples, a person’s greater perception of their partner’s active–constructive responses was associated with several positive relational and individual outcomes for that person (e.g., greater relationship satisfaction and positive emotions; Gable et al., 2004; Kashdan et al., 2013; Monfort et al., 2014) and their partner (Pagani et al., 2020). Conversely, a person’s greater perception of their partner’s passive–constructive, active–destructive, and passive–destructive responses was negatively correlated with that person’s commitment, daily satisfaction, and positive emotions (Donato et al., 2014; Gable et al., 2004). The majority of those studies found no gender differences, but a few suggest that perceived responses to capitalization attempts are more strongly associated with women’s relationship outcomes (e.g., Pagani et al., 2020). Most studies did not examine participants’ perception of their own responses in a context of capitalization, even though recent studies demonstrate that emitting active–constructive responses was also associated with individual and relational benefits (Kashdan et al., 2013; Hershenberg et al., 2016; Monfort et al., 2014). Also, most did not adopt observational designs, which allow for the measurement of participants’ own and their partner’s responses, with minimal retrospective bias and using objective external observer ratings.

Only one study examined associations between responses to capitalization attempts and sexuality using a cross-sectional, observational, and self-report design among 178 heterosexual couples (Birnbaum et al., 2016). Results showed that for women, their partner’s responsiveness following the disclosure of a positive event, as observed by external coders, was positively associated with their own sexual desire. For both men and women, their perception of their partner’s responsiveness was positively associated with their own sexual desire. Although novel, this study involved young couples, most of whom did not live together (i.e., may not experience declines in sexual well-being), focused on sexual desire exclusively, and did not assess partner effects (e.g., the association between a person’s responsiveness and their partner’s sexual desire). Lastly, the self-report questionnaires and observational method were not adapted to the disclosure of positive events. Instead, a general measure of partner responsiveness (e.g., behaviors that signal understanding, validation, and caring) was used. Yet, the reactions that are thought to be responsive following the disclosure of a negative event are not necessarily the same as those following the disclosure of a positive event. For example, a simple nod might be considered responsive in a negative disclosure context, whereas the same nod might be considered dismissive in a positive disclosure context (Gable et al., 2012). Thus, the failure to adapt the measure to the specific context can limit the conclusions that can be drawn concerning responses to capitalization attempts and sexual well-being.

### Current study

The current study aimed to examine associations between one’s perception of their own and their partner’s responses as well as observed responses (assessed by external coders) to capitalization attempts in the laboratory, and sexual well-being (i.e., sexual satisfaction, distress, and function) in an inclusive sample of same- and mixed-gender cohabiting couples. Given changes in couples’ sexual well-being over time (e.g., Gunst et al., 2017; McNulty et al., 2016), relationship length was included as a covariate. We expected that a person’s greater perception of their own and their partner’s active–constructive responses, as well as greater active–constructive responses observed in that person, would be associated with the person and their partner’s greater sexual satisfaction and function, and lower sexual distress. We also expected that a person’s greater perception of their own and their partner’s passive–constructive, active–destructive, and passive–destructive responses, as well as greater passive–constructive, active–destructive, and passive–destructive responses observed in that person, would be associated with their own and their partner’s lower sexual satisfaction and function, and greater sexual distress.

## Method

### Participants

A convenience sample of 151 couples was recruited through advertisements on social media and websites (e.g., blogs, Facebook), university listservs, flyers displayed in public places and by word of mouth. In addition, couples who already participated in previous studies led by our research team and who consented to be recontacted for future research were invited to participate. Recruitment was conducted in two Canadian cities between May 2019 and January 2020.

Interested couples were screened for eligibility using a structured telephone interview. The inclusion criteria were the following: (a) be in a monogamous relationship and cohabiting for at least 1 year, (b) understand written and spoken French or English, (c) be at least 18 years old, and (d) have already been sexually active at one time in their life (not specific to their current relationship). The exclusion criteria were as follows: (a) presence of a self-reported major medical or psychiatric problem, (b) pregnancy, breastfeeding, or parents of a child of less than 1 year, (c) medication or drugs that alter sexuality significatively, and (d) currently being treated specifically for sexual problems or considering starting this type of treatment during the study. Of the 570 couples who contacted us to participate in the study, 304 (53.3%) declined to participate and 108 (18.9%) were ineligible after screening. Of the 158 eligible couples, four (2.5%) failed two out of three attention-testing questions in the baseline survey and three (1.9%) did not complete the baseline survey and thus, were not invited to participate in the laboratory session. The final sample included 151 couples (302 participants). Among those, 145 (48%) identified as cis men, 150 (49.7%) identified as cis women, and 7 (2.3%) identified as non-binary, queer, or gender fluid. Thus, the sample included eight (5.3%) women–women couples, seven (4.6%) men–men couples, 129 (85.4%) women–men couples, and seven (4.6%) couples that included one partner who identified as non-binary, queer, or gender fluid.

Participants were aged between 18 and 63 years old (*M* = 31.92, *SD* = 9.06, *Median* = 30.00). On average, participants had 16.30 years of education (*SD* = 2.76) and 19.5% of the sample reported being full-time students and 3% part-time students. The relationship length ranged from one to 37 years (*M* = 6.52, *SD* = 6.07). Most participants reported an annual income under $59,999 (77.8%, *n* = 235), 12.3% reported an annual income between $60,000 and $79,000 (*n* = 37) and 9.9% reported an annual income over $80,000 (*n* = 30). Almost half of the sample indicated that their cultural identity was English Canadian (47.7%, *n* = 144), 37.7% identified as French Canadian (*n* = 114), 5.3% as Western European (*n* = 16), and 9.3% as other cultural identities (*n* = 28; First Nations, American, Eastern European, Australian, Middle Eastern, Latin American/South American, Caribbean). Regarding sexual orientation, 69.2% identified as heterosexual (*n* = 209), 8.3% as lesbian or gay (*n* = 25), 7.0% as heteroflexible (*n* = 21), 7.9% as bisexual (*n* = 24), 0.7% as homoflexible (*n* = 2), 1.7% as queer (*n* = 5), 3.0% as pansexual (*n* = 9), 0.7% as asexual (*n* = 2), 0.7% selected the “other” option (demisexual; gray-asexual biromantic (*n* = 2), 0.7% were questioning their sexual orientation (*n* = 2), and 0.3% did not want to answer (*n* = 1). As for relationship status, 45 (30.0%) couples were married, and 106 (70.0%) were common-law partners.

### Procedure

All 151 couples attended a two-hour laboratory session to participate in filmed discussions. One week before their appointment, each partner received a secure link by email hosted by Qualtrics Research Suite, where they provided informed written consent and completed self-report measures assessing sociodemographic characteristics as well as sexual satisfaction, distress, and function. All procedures were approved by both university’s Institutional Review Boards. Each couple received a compensation of CAN $20 in Amazon gift card for the baseline survey and CAN $100 for the laboratory session.

During the laboratory session, each member of the couple was invited to disclose a positive personal event (big or small) that neither had involved nor had been shared with the partner and that had occurred in the past month.

The discussion task lasted 8 minutes and members of each couple took turns being a speaker for 4 minutes and a listener for 4 minutes alternately. Immediately after this discussion, each partner completed self-report questionnaires assessing their experience, including the importance (significance) of the event they disclosed on a scale of 1 (*not very important*) to 6 (*extremely important; M* = 4.00, *SD* = 1.29) and the representativeness of the discussion they just had compared to a typical discussion that they would have at home on a scale of 0 (*not at all*) to 5 (*extremely*; *M* = 4.09, *SD* = 0.67). On average, participants perceived their discussion to be realistic and based on a significant positive event. Trained observers later coded the videotapes of this discussion (see coding procedure below). This discussion task was based on past standardized couple observation studies (e.g., Bois et al., 2016; Gable et al., 2006; Kashdan et al., 2013). The data and syntaxes can be obtained at: https://osf.io/29rjh/?view_only=36229505849547bd9059c2fec5faa768.

### Baseline measures

*Sexual Satisfaction:* To assess partners’ satisfaction with their current sexual relationship, we used the Global Measure of Sexual Satisfaction (GMSEX; Lawrance & Byers, 2010). Participants were asked to describe their sexuality overall in the last 6 months by rating five 7-point bipolar scales: “good-bad,” “pleasant-unpleasant,” “positive-negative,” “satisfying-unsatisfying,” and “valuable-worthless.” Scores range from 5 to 35, with higher scores indicating greater sexual satisfaction. Lawrance and Byers (2010) demonstrated excellent psychometric properties for this measure. The internal consistency in the present sample was excellent (α = .92).

*Sexual Distress:* Participants reported on their sexual distress using the Female Sexual Distress Scale-Revised (FSDS-R; Derogatis et al., 2002), also validated in men (Santos-Iglesias et al., 2018). This 13-item scale assesses how often a sexual problem has bothered them or caused them distress over the previous month (e.g., “How often did you feel stressed about sex?”). Items are answered on a 5-point Likert-type scale ranging from 0 (*never*) to 4 (*always*). Scores range from 0 to 52, with higher score indicating greater sexual distress. The revised Female Sexual Distress Scale has good psychometric properties (Derogatis et al., 2002) and the internal consistency in the present sample was excellent (α = .93).

*Sexual Function:* Participants were asked to choose between completing the “male-bodied” or the “female-bodied” measure of sexual function. For the male-bodied measure, the International Index of Erectile Function (IIEF; Rosen et al., 1997) was used. This measure of 15 items assesses five domains: erectile function, orgasmic function, sexual desire, intercourse satisfaction, and overall satisfaction. Participants answered on a 5-point Likert-type scale, with higher scores indicating greater sexual function. This measure has good psychometric properties (Rosen et al., 1997) and its internal consistency in the present sample was good (α = 0.79). For the female-bodied measure, the Female Sexual Function Index (FSFI; Rosen et al., 2000) was used. This measure of 19 items assesses six domains: sexual desire, arousal, lubrication, orgasm, satisfaction, and pain. Participants answered on a 5-point Likert-type scale, with higher scores indicating greater sexual function. Rosen et al. (1997) demonstrated good psychometric properties for this measure and its internal consistency in the present sample was excellent (α = 0.92). To be able to interpret scores in the same way for all participants, total scores of the FSFI were rescaled. We transformed the original FSFI score to an adjusted FSFI score that had a comparable range to the IEFF through this formula: [(*χ* – 2) x (75/34)] (Corsini-Munt et al., 2017). Thus, total scores range from 15 to 75, with higher scores indicating greater sexual function. For the FSFI and IIEF, items of participants who had no sexual activity in the last 4 weeks were recoded as “missing” to avoid skewing the score toward dysfunction (Meyer-Bahlburg & Dolezal, 2007).

### Post-discussion measures

*Perceived Partner Responses:* To assess participants’ perception of their partner’s responses during the discussion, the 12-item Perceived Responses to Capitalization Attempts Scale (PRCA; Gable et al., 2004) was used. This measure assesses the four types of responses with three items each: active–constructive (e.g., “I got the sense that my partner was even more happy and excited than I am”), passive–constructive (e.g., “My partner is usually silently supportive of the good things that occur to me”), active–destructive (e.g., “My partner found a problem with it”), and passive–destructive (e.g., “My partner seemed disinterested”). Each item is based on a 7-point Likert scale ranging from 1 (*not at all true*) to 7 (*very true*). The score for each subscale vary from 1 to 7 with higher scores indicating greater responses from the subscale. This measure has good psychometric properties (Gable et al., 2004, 2006, 2012) and the internal consistency in the present sample was acceptable for each subscale (active–constructive: α = .72; passive–constructive: α = .72; active–destructive: α = .70; passive–destructive: α = .85)

*Self-Reported Responses:* In order to assess participants’ own self-report of their responses during the discussion, the Perceived Responses to Capitalization Attempts Scale (PRCA; Gable et al., 2004; 12 items) was adapted by changing pronouns in each item. For example, the item “My partner found a problem with it” was modified to “I found a problem with it.” This measure assesses the four types of responses with the same scale as described above. Internal consistency in the present sample was acceptable for each subscale (active–constructive: α = .69; passive–constructive: α = .81; active–destructive: α = .76; passive–destructive: α = .77).

### Observed responses

Partners’ responses during the discussion were rated independently by two trained coders based on the *Interpersonal Process Model of Intimacy* (Reis & Shaver, 1988), adapted to the capitalization context (Gable et al., 2004; Kashdan et al., 2013). Coders received descriptions of the four types of partner responses to sharing of positive events developed by Kashdan et al. (2013). They received the instructions to watch the videos at least twice and not to rate two partners of the same couple one after the other in order to reduce possible bias. Coders rated the degree to which the partner’s responses matched each of the four types of responses using a 6-point Likert scale (0 = *absolutely no match* to 5 = *very good match*; Kashdan et al., 2013). Inter-rater reliability, estimated through intraclass correlations (ICC), was excellent for each subscale (active–constructive: ICC = .90 (95% CI .87 to .92); passive–constructive: ICC = .94 (95% CI .93 to .95); active–destructive: ICC = .88 (95% CI .84 to .90); and passive–destructive: ICC = .86 (95% CI .82 to .89)). We averaged the two coders’ ratings to obtain a total score for each subscale.

### Data analytic strategy

Descriptive and bivariate correlation analyses were performed using SPSS 26.0. The hypotheses were tested using Mplus 8.0 (Muthén & Muthén, 1998–2017). Associations between responses (i.e., perceived, self-reported, and observed) and sexual outcomes (i.e., sexual satisfaction, sexual distress, and sexual function) were examined using path analysis within an actor-partner interdependence model (APIM; Kenny et al., 2006). APIM analyses account for the interdependence of dyadic data and examine simultaneously both actor effects (e.g., the association between a person’s active–constructive responses and their own sexual satisfaction) and partner effects (e.g., the association between a person’s active–constructive responses and their partner’s sexual satisfaction). Dyads were considered as indistinguishable as this sample includes both same- and mixed-gender couples, precluding using sex/gender as the distinguishing variable. Thus, each member of the couple was randomly assigned to “partner 1” and “partner 2” and all parameters were constrained to be equal between partners (i.e., variances, actor effects, partner effects, means, and intercepts; West, 2013). Three APIMs were tested; one model for perceived partner responses, one for self-reported responses, and one for observed responses. All three sexual outcomes were entered simultaneously as dependent variables and relationship length was included as a covariate in each model. To examine gender differences in the associations between responses (perceived, self-reported, and observed) and sexual outcomes, the interactions between a person’s responses and their own gender (men = −0.50, women = 0.50) were added to the models. Only when interactions with gender were tested, non-binary, queer, or gender fluid individuals were excluded due to the small sample size (*n* = 7). When an interaction term was significant, simple slope tests were used to report the associations for women and men. All analyses were performed with the maximum likelihood parameter estimates with robust standard errors and chi-square test (MLR) and missing data were handled using Full Information Maximum Likelihood (FIML; Muthén & Muthén, 1998–2017). Commonly used goodness-of-fit indices were used to evaluate models (Hu & Bentler, 1999; Kline, 2015; Schermelleh-Engel et al., 2003): comparative fit index (CFI; ≥ .90 acceptable; ≥ .95 good), root mean square error of approximation with its 90% confidence interval (RMSEA; ≤ .08 adequate; ≤ .06 good), and standardized root mean square residual (SRMR; ≤ .10 adequate; ≤ .08 good).

## Results

### Descriptive statistics

Means (*M*), standard deviations (*SD*), and correlations between the study variables are presented in Table 1.

**Table 1.** Descriptive statistics and correlations among perceived, self-reported, and observed responses to capitalization attempts and sexual satisfaction, sexual distress, and sexual function.

	*n*	*M* (*SD*)	Range	1	2	3	4	5	6	7	8	9	10	11	12	13	14	15
1. Perceived AC responses	298	5.25 (1.10)	1.67–7	**.04**	−.35**	^−^.23**	^−^.53*	.31**	^−^.02	^−^.08	^−^.11	^−^.02	.06	^−^.09	^−^.09	.20**	^−^.11	.11
2. Perceived PC responses	298	3.64 (1.57)	1–7	^−^.03	.28**	.18**	.20**	.01	.61**	.07	.06	^−^.07	.13*	.03	.13*	^−^.15*	.10	^−^.06
3. Perceived AD responses	298	1.30 (0.64)	1–5.33	^−^.02	.08	**.04**	.31**	^−^.06	.16**	.31**	.24**	^−^.02	.06	^−^.02	.04	^−^.15**	.03	.04
4. Perceived PD responses	298	1.30 (0.67)	1–6	^−^.07	.05	.14*	.15**	^−^.06	.03	.23**	.27**	.11	^−^.12*	.09	.01	^−^.20**	.17**	^−^.10
5. Self-reported AC responses	298	4.96 (1.03)	1.67–7	.28**	^−^.24**	^−^.05	^−^.12	^**−**^ **.01**	^−^.28**	^−^.15**	^−^.41**	.30**	^−^.16**	.03	^−^.12*	.22**	^−^.12*	.04
6. Self-reported PC responses	298	3.67 (1.71)	1–7	^−^.19**	.43**	.09	.11	^−^.04	.28**	.09	.22**	^−^.27**	.26**	^−^.02	.04	^−^.13*	.08	^−^.01
7. Self-reported AD responses	298	1.27 (0.61)	1–5	^−^.10	.13*	.33**	.09	^−^.04	.11	.14*	.42**	^−^.04	^−^.03	.26**	.02	^−^.21**	.12*	^−^.12*
8. Self-reported PD responses	298	1.34 (0.67)	1–4.33	^−^.26**	.14*	.13*	.28**	^−^.02	^−^.01	.05	**.02**	^−^.07	.00	.02	.08	^−^.22**	.22**	^−^.06
9. Observed AC responses	292	2.90 (1.03)	0–5	.32**	^−^.31**	^−^.04	^−^.06	.01	^−^.03	^−^.02	.10	**.06**	^−^.79**	^−^.15**	^−^.31**	.14*	^−^.09	^−^.06
10. Observed PC responses	292	0.88 (1.18)	0–4	^−^.17**	.25**	^−^.04	^−^.01	^−^.07	.07	.02	^−^.09	^−^.03	**.06**	^−^.13*	^−^.07	^−^.06	.05	.08
11. Observed AD responses	292	0.23 (0.55)	0–4	^−^.01	.07	.23**	.05	^−^.05	.14*	.03	.09	^−^.08	^−^.00	**.07**	.15**	^−^.12*	.14*	^−^.20**
12. Observed PD responses	292	0.41 (0.71)	0–4	^−^.14*	.06	^−^.00	.07	^−^.02	.07	^−^.01	^−^.05	^−^.08	^−^.05	.09	.13*	^−^.08	^−^.05	.07
13. Sexual satisfaction	302	29.50 (5.94)	5–35	.20**	^−^.17**	^−^.01	^−^.07	.07	^−^.11	^−^.01	^−^.05	.14*	^−^.10	^−^.02	^−^.04	.33**	^−^.52**	.55**
14. Sexual distress	302	12.30 (9.75)	0–45	^−^.08	.08	.08	.14*	^−^.06	.11	^−^.01	.09	^−^.10	.13*	.02	^−^.06	^−^.39**	.39**	^−^.64**
15. Sexual function	295	61.84 (9.53)	24.27–75	−.02	.02	^−^.10	^−^.01	.07	^−^.09	^−^.02	^−^.06	.07	^−^.10	.02	.06	.27**	^−^.22**	.11*

*Note.* AC = active–constructive; PC = passive–constructive; AD = active–destructive; PD = passive–destructive. Correlations above the diagonal are between each of the actor variables and correlations along (in bold) and below the diagonal are between the actor and partner variables.

^*^ *p* < .05, ^**^ *p* < .010, ^***^ *p* < .001. *M* = mean; *SD* = standard deviation.

### Associations between perceived partner responses and sexual outcomes

**Figure 1.**
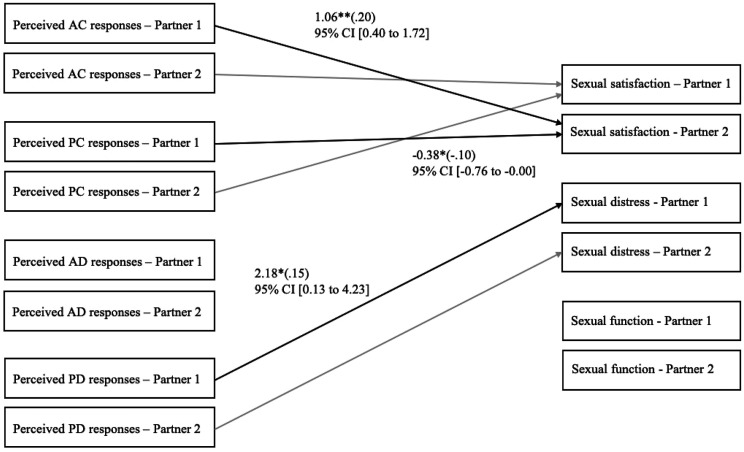
Associations between perceived responses to capitalization attempts and sexual satisfaction, distress, and function of both partners, after controlling for relationship length. To simplify presentation, only significant unstandardized coefficients (standardized coefficients) are depicted in this figure. These associations were not significantly different between men and women. Considering that these are indistinguishable dyads, actor and partner associations are constrained to be equal, meaning that associations for Partner 2 are the same as the ones for Partner 1. Thus, we depicted associations for Partner 2 in light gray. AC = active–constructive. PC = passive–constructive. AD = active–destructive. PD = passive–destructive. CI = confidence intervals. * *p* < .05. ** *p* < .01.

Firstly, we examined the associations between perceived partner responses and sexual satisfaction, distress, and function controlling for relationship length. Results are presented in Figure 1 and showed that a person’s greater perception of their partner’s active–constructive responses was associated with their partner’s greater sexual satisfaction. A person’s greater perception of their partner’s passive–constructive responses was associated with their partner’s lower sexual satisfaction. Finally, a person’s greater perception of their partner’s passive–destructive responses was associated with that person’s greater sexual distress. This model provided good fit indices: χ^2^ (63) = 62.65, *p* = .489; RMSEA = .00, 90% CI [.00, .05]; CFI = 1.00; SRMR = .09, and explained 11.2% of the variance in sexual satisfaction, 5.2% of the variance in sexual distress, and 3.4% of the variance in sexual function. We then added the interactions between one’s perception of their partner’s responses and one’s own gender (men = −0.50, women = 0.50; *n* = 144) to examine if the actor and partner effects were significantly different between men and women. All interactions were nonsignificant; thus, all associations were similar between women and men.

### Associations between self-reported responses and sexual outcomes

**Figure 2.**
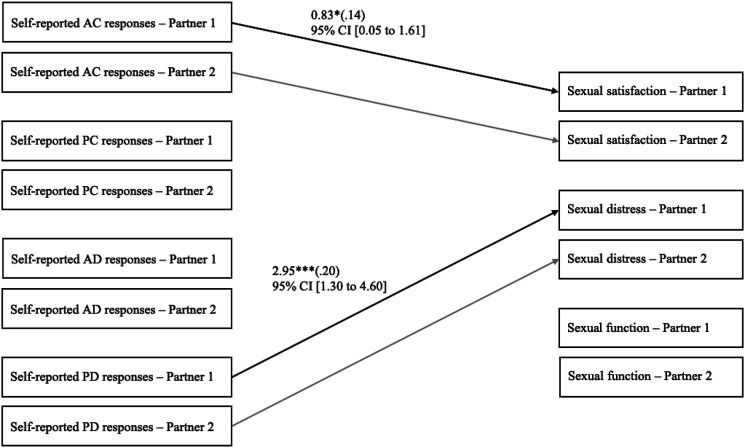
Associations between self-reported responses to capitalization attempts and sexual satisfaction, distress, and function of both partners, after controlling for relationship length. To simplify presentation, only significant unstandardized coefficients (standardized coefficients) are depicted in this figure. These associations were not significantly different between men and women. Considering that these are indistinguishable dyads, actor and partner associations are constrained to be equal, meaning that associations for Partner 2 are the same as the ones for Partner 1. Thus, we depicted associations for Partner 2 in light gray. AC = active–constructive. PC = passive–constructive. AD = active–destructive. PD = passive–destructive. CI = confidence intervals. * *p* < .05. ** *p* < .01. *** *p* < .001.

Secondly, we examined the associations between self-reported responses and sexual satisfaction, distress, and function controlling for relationship length. Results are presented in Figure 2 and showed that a person’s reported greater active–constructive responses were associated with their own greater sexual satisfaction. A person’s reported greater passive–destructive responses were associated with their own greater sexual distress. This model provided good fit indices: χ^2^ (63) = 64.37, *p* = .429; RMSEA = .01, 90% CI = [.00, .05]; CFI = 1.00; SRMR = .09, and explained 10.0% of the variance in sexual satisfaction, 7.3% of the variance in sexual distress, and 2.5% of the variance in sexual function. We then added the interactions between a person’s self-reported responses and one’s own gender (men = −0.50, women = 0.50; *n* = 144). The association between a person’s active–constructive responses and their partner’s sexual function was significantly different between women and men as the interaction term was significant, b (SE) = 0.52 (0.24), *p* = .032; 95% CI = [0.05, 1.00]; β = .15. However, the simple slope test indicated that the association was nonsignificant in both women, b (SE) = 0.05 (0.46), *p* = .919; 95% CI = [−0.86, 0.95]; β = .05 and men, b (SE) = −0.48 (0.53), *p* = .364; 95% CI = [−1.51, 0.55]; β = −.10. No other gender differences were found.

### Associations between observed responses and sexual outcomes

**Figure 3.**
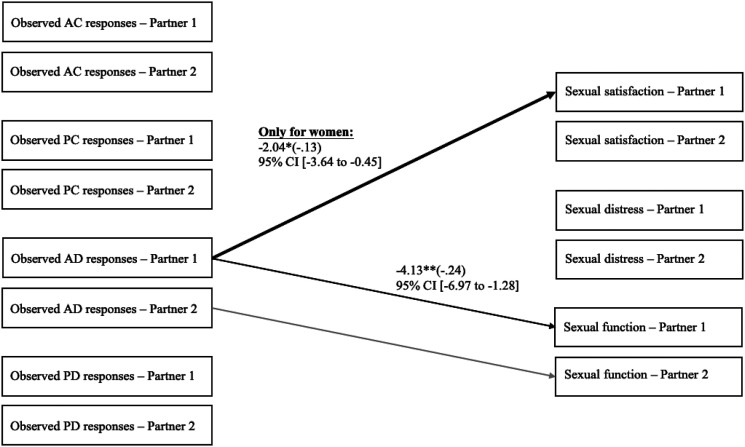
Associations between observed responses to capitalization attempts and sexual satisfaction, distress, and function of both partners, after controlling for relationship length. To simplify presentation, only significant unstandardized coefficients (standardized coefficients) are depicted in this figure. Considering that these are indistinguishable dyads, actor and partner associations are constrained to be equal, meaning that associations for Partner 2 are the same as the ones for Partner 1. Thus, we depicted associations for Partner 2 in light gray. The bold line represents the only association that is different between men and women (*n* = 144). AC = active–constructive. PC = passive–constructive. AD = active–destructive. PD = passive–destructive. CI = confidence intervals. * *p* < .05. ** *p* < .01.

Finally, we examined the associations between observed responses and sexual satisfaction, distress, and function controlling for relationship length. As presented in Figure 3, results showed that a person’s greater active–destructive responses, as observed by external coders, were associated with that person’s lower sexual function. This model provided good fit indices: χ^2^ (63) = 92.70, *p* = .009; RMSEA = .06, 90% CI [.03, .08]; CFI = 0.91; SRMR = .09, and explained 7.8% of the variance in sexual function, 5.7% of the variance in sexual distress, and 4.7% of the variance in sexual satisfaction. We then added the interactions between participants’ observed responses and their own gender (men = −0.50, women = 0.50; *n* = 144). The association between a person’s active–destructive responses, as observed, and their own sexual satisfaction was significantly different between women and men as the interaction term was significant, b (SE) = −2.55 (0.85), *p* = .003; 95% CI = [−4.22, −0.88]; β = −.13. The simple slope test indicated that women’s active–destructive responses, as observed, were related to their own lower sexual satisfaction, b (SE) = −2.04 (0.81), *p* = .012; 95% CI = [−3.64, −0.45]; β = −.13, whereas in men, this association was non-significant, b (SE) = 0.51 (0.86), *p* = .556; 95% CI = [−1.18, 2.20]; β = −.01. No other gender differences were found.

## Discussion

This dyadic study examined associations between perceived, self-reported, and observed responses to capitalization attempts and sexual satisfaction, distress, and function in same- and mixed-gender cohabiting couples. Results indicated that higher levels of self-reported and partner-perceived active–constructive responses during the discussion were associated with one’s own greater sexual satisfaction. Conversely, higher levels of perceived passive–constructive responses from one’s partner were associated with one’s own lower sexual satisfaction, and higher levels of self-reported and perceived passive–destructive responses were associated with greater sexual distress. Finally, higher levels of observed active–destructive responses were associated with one’s own lower sexual function, and in women, with their lower sexual satisfaction. Findings support the *Sexual Crucible Model* (Schnarch, 2009) as active–constructive responses were linked to greater sexual well-being, whereas passive–constructive, passive–destructive, and active–destructive responses were associated with poorer sexual outcomes.

### Active–constructive responses

Consistent with our hypothesis, findings indicated that the more a person self-reported as having demonstrated enthusiasm and genuine interest in their partner’s positive event, and the more the partner perceived it as such, the greater this person’s reported sexual satisfaction. Being able to be responsive to the partner in that particular context is a demonstration of the capacity to encourage their individuality, while remaining intimately connected (Gable et al., 2006; Hadden et al., 2015; Reis et al., 2010), considering that the partner is disclosing a positive event outside the relationship. Thus, results support the *Sexual Crucible Model* (Schnarch, 2009), according to which intimacy is central to creating a satisfying sexual and romantic relationship and is defined as the ability to join two opposing drives: individuality and togetherness. Recognizing the other as different from oneself involves tolerating feelings of vulnerability, which are at the heart of facilitating closeness between partners and inherent to satisfying sexuality (Schnarch, 2009). In contrast to our hypothesis, active–constructive responses were not associated with less sexual distress and function. However, it should be noted that sexual satisfaction is not the same as the simple absence of sexual difficulties or sexual distress, as it refers to a general subjective impression of a positive and satisfying sex life (Rosen & Bachmann, 2008). Indeed, studies increasingly show that positive contexts have unique predictive value in assessing positive outcomes, above and beyond negative factors (Graber et al., 2011).

### Passive–constructive responses

As expected, participants’ greater perception that their partner had been quiet and silently supportive but attentive and/or interested during the discussion was associated with their partner’s lower levels of sexual satisfaction. This result supports the *Sexual Crucible Model* and also underlines that behaviors associated with positive outcomes in times of distress are not necessarily adaptive following the disclosure of a positive event (Donato et al., 2014; Gable et al., 2004). It is possible that a person who does not show much enthusiasm and does not actively encourage their partner to elaborate on their positive event, but still shows some interest and attention toward it, might tolerate the partner’s individuality, but not particularly cherish and promote it. Consequently, although this type of response is not associated with sexual distress or sexual function, the results suggest that simply tolerating a partner’s individuality may not, in and of itself, be associated with a satisfying sexuality. Both partners might tolerate that each has a life outside of the relationship, but be less likely to share those personal experiences with each other. This might limit the possibility of introducing vitality and novelty into the relationship and of seeing the other in a new light. In turn, this limitation might alter the possibility to deepen the level of intimacy, resulting in less sexual satisfaction (Kuten, 1976; Schnarch, 2009).

### Active–destructive responses

When a person was observed by external coders as undermining and/or denying the positive nature of the event, that person reported lower sexual function. Also, when women were observed by external coders as undermining and/or denying the positive nature of the event, they reported lower levels of sexual satisfaction. These results are consistent with our hypothesis. Indeed, a person who emits active–destructive responses might have a harder time accepting their partner’s individuality (Gable et al., 2006; Hadden et al., 2015; Reis et al., 2010). In fact, the context of capitalization attempts in which partners are asked to disclose a positive event that did not include the partner is unique as it exposes the notion of separation between the partners: the other exists and is experiencing positive events outside of the relationship. When differentiation is not acquired in a couple, this context could be threatening for partners as they are confronted with their separateness, and they might actively try to dismiss the event and the partner’s enthusiasm toward it. As the *Sexual Crucible Model* posits, when there is no differentiation between partners, they are in a state of emotional fusion, which could hinder sexual well-being (Perel, 2007; Schnarch, 2009). This result is in line with that of Burri et al. (2014) who found that the inability to maintain a sense of self in the presence of intimate others was the strongest predictor of sexual problems in a sample of women. Findings are also consistent with those of a qualitative study in which married women reported that one of the main reasons for their decreased desire was lack of individuation (Sims & Meana, 2010). However, active–destructive responses were not associated with sexual satisfaction in men. Although contrary to our hypothesis, this result coheres with research that suggests women’s sexuality is more strongly influenced by relational factors relative to men’s (Basson, 2002; Peplau, 2003). In addition, only the observed active–destructive responses (not perceived or self-reported) were related to sexual outcomes. The difficulty for individuals to perceive themselves and their partners as having emitted active–destructive responses, possibly to protect their positive self- and partner-illusions (Conley et al., 2009; Luo & Snider, 2009), might explain this finding. From a methodological standpoint, the study coders’ grid assessed subtler non-verbal behaviors compared to the perceived and self-reported questionnaires.

Moreover, it is relevant to wonder why the only independent variable associated with sexual function is the observation of active–destructive responses. It should be noted that the sexual function questionnaire primarily measured the frequency or level of difficulty of each area of sexual function; it is therefore closer to behaviors than the two other outcomes, sexual satisfaction and sexual distress, which are more focused on the emotional aspects of sexuality. Thus, the assessment of sexual function as well as the assessment of the observed active–destructive responses by external coders are two rather “objective” measures, which could possibly explain why an association emerged only for these two variables.

### Passive–destructive responses

Consistent with our hypotheses, the more a person perceived themselves or their partner as uninterested and self-focused following the disclosure of a positive event, the more this person reported sexual distress. This is the only type of response associated with sexual distress and, when perceived and not only emitted, associated with a sexual outcome. Passive–destructive responses refer to a complete indifference, a “nonresponse” to the event. Pagani et al. (2020) showed that passive–destructive responses were associated with less emotional closeness compared to the active–destructive and passive–constructive responses. This type of response can be seen as the opposite of emotional fusion, that is, emotional detachment (Perel, 2007). Passive–destructive responses can be likened to the stage of romantic disengagement, characterized by an indifference toward the partner, lack of positive emotions, but also few displays of negative emotions. This stage is often associated with less intimacy and trust, and a greater likelihood of relationship break up and dissatisfaction (Abbasi & Alghamdi, 2017). Yet, too much distance between partners to a point of feeling disconnected emotionally can be harmful to couples’ sexual well-being (Perel, 2007; Schnarch, 2009). This is also consistent with studies demonstrating high correlations between dissatisfaction with the relationship and sexuality-related negative affect (Blumenstock & Papp, 2017; McNulty et al., 2016).

The overall pattern of results, whereby self-reported responses were more strongly associated with participants’ sexual outcomes than perceived ones, suggests that perceiving a partner’s responses to capitalization attempts is less important to one’s own sexuality than emitting such responses toward the partner, that is, being able (or not) to acknowledge and promote the partner’s individuality. These results are novel, considering that in the study of intimacy in general as well as in the context of capitalization attempts, the emphasis is often placed on the perception of partner responses (e.g., perceived partner responsiveness; Gable et al., 2006; Pagani et al., 2020). Indeed, most studies did not examine participants’ perception of their own responses and most did not use an observational design. Thus, results of this study underline the importance for future studies to include the assessment of one’s own responses toward the partner.

Moreover, it should be noted that we found only one partner effect, contrary to our hypotheses. This is consistent however with the meta-analysis using machine learning across 43 dyadic longitudinal datasets from 29 laboratories, which found that actor effects predicted two to four times more variance in relationship quality than partner effects (Joel et al., 2020).

### Strengths and limitations

This study has some limitations. First, the cross-sectional design precludes any conclusion concerning the directionality of associations. Future research should examine sexual well-being using a longitudinal design to assess directionality, and to investigate how responses to capitalization attempts predict different trajectories of couples’ sexual well-being over time (Leonhardt et al., 2021). However, results are consistent with theoretical-clinical models (Perel, 2007; Schnarch, 2009; Kuten, 1976). Furthermore, a recent daily diary study examining associations between intimacy and sexual satisfaction, pain, and sexual function in couples coping with genito-pelvic pain, statistically tested directionality between intimacy and sexuality. The results showed that it was indeed intimacy that predicted sexuality outcomes (Bergeron et al., 2021). Second, the discussion taking place in the laboratory might have limited ecological validity, although on average, participants perceived their discussion to be realistic. Third, the sample lacked ethnic diversity and we did not ask participants if they had any disabilities. Moreover, it should be noted that sexuality is multidetermined and even though this study shows that responses to capitalization attempts are linked with couples’ sexuality, the explained variance for each sexual outcome remains low; several other variables could potentially explain variations in couples’ sexual well-being. However, it is notable that partners’ responses in one very specific in-lab interaction relate to their sexual well-being. In addition, since couples’ average age was in the early thirties, participants reported, on average, relatively low levels of sexual difficulties and distress, and the means and standard deviations of self-reported, perceived, and observed active–destructive and passive–destructive responses were quite low (see Table 1). Thus, the limited variance in those variables might explain certain non-significant associations. Future research should study longer-term couples to better identify factors associated sexual difficulties. Despite these limitations, this study was the first to our knowledge to investigate associations between responses to capitalization attempts and couples’ sexual well-being. One of its major strengths was the use of external coders as well as self-report questionnaires completed immediately following the discussion. This method allowed us to measure participants’ perception of both their own and their partner’s responses with minimal retrospective bias and greater objectivity. Finally, this study was conducted among cohabiting couples and included participants of all sexual orientations and gender identities.

## Conclusions and implications

Findings showed that the responses following the disclosure of a positive event were associated with various facets of couples’ sexual well-being, independent of relationship length. Overall, results support the *Sexual Crucible Model* (Schnarch, 2009), as active–constructive responses were associated with greater sexual well-being, and the other responses, with poorer sexual well-being. Since the findings were not consistent across sexual outcomes, they reinforce the idea that sexual satisfaction, distress, and function are distinct constructs and underline the multidimensional aspect of sexual well-being.
